# Schoolteachers Teach First Aid and Trauma Management to Young Primary School Children: An Experimental Study with Educational Intervention

**DOI:** 10.3390/children10061076

**Published:** 2023-06-19

**Authors:** Eleana Tse, Katerina Plakitsi, Spyridon Voulgaris, George A. Alexiou

**Affiliations:** 1 Department of Neurosurgery, School of Medicine, University of Ioannina, 45500 Ioannina, Greece; e.tse@uoi.gr (E.T.); svoulgar@uoi.gr (S.V.); 2 Department of Early Childhood Education, School of Education, University of Ioannina, 45110 Ioannina, Greece; kplakits@uoi.gr

**Keywords:** children, first aid training, school program, schoolteachers, primary school

## Abstract

Objectives: The primary objective of this pilot study was to examine the effectiveness of teaching first aid to 6–8-year-old children within their primary school setting. The study aimed to address two key research questions: (1) Can children of this age group acquire first aid and trauma management skills from their schoolteachers? (2) How long do children retain the acquired first aid knowledge? Methods: A pilot experimental study with an educational intervention was conducted in a single primary school in Greece. A total of 60 schoolchildren aged 6–8 years were randomly selected for participation, with 30 children assigned to the training group and 30 children assigned to the control group, which did not receive any intervention. To assess the children’s understanding of first aid, a specialized questionnaire was administered to all children one day before the training, as well as to the training group one day after the training, and at two and six months following the training. Results: Prior to the training, there were no significant differences in first aid knowledge between the children in the training group and those in the control group. However, one day after the training, the trained children demonstrated significantly higher scores (*p* < 0.05) compared to the control group. Over time, the first aid knowledge of the trained children gradually declined at the two- and six-month follow-up assessments, although it remained higher than their pre-training level. Conclusions: First aid training provided by their teachers improved the knowledge of 6–8-year-old primary school children in first aid and trauma management.

## 1. Introduction

The World Health Organization (WHO) recognizes the importance of educating individuals of all ages on essential health topics, including the ability to respond to emergencies [1]. First aid plays a critical role in providing immediate care and attention to injured or ill individuals before professional medical help becomes available. First aid courses aim to equip members of the public with the skills to handle health emergencies using basic knowledge and techniques, without relying on specialized medical expertise or technology. In line with the WHO recommendations, researchers are exploring the effectiveness of first aid training for schoolchildren as a means to enhance first aid and trauma management knowledge within the broader population [2,3,4,5,6].

While previous studies have primarily focused on specialized first aid training for children above the age of ten [2], a few investigations have examined first aid education for younger children [7]. Notably, Bollig and colleagues [4] found that 4–5-year-old children could grasp and apply basic first aid principles. Banfai and colleagues [3] reported significant improvements in knowledge, attitudes, and skills, with retention of first aid information for up to 15 months following educational interventions in children aged 7–14 years. Furthermore, Mohajervatan and colleagues [8] demonstrated that young children are capable of learning emergency first aid activities, including calling an ambulance, managing an unconscious patient, and controlling severe bleeding.

This pilot study aims to assess the effectiveness of schoolteachers in teaching first aid to 6–8-year-old children in a Greek primary school. The study aims to address the following research questions:

1. Can children of this age group acquire first aid and trauma management skills from their schoolteachers?
2. How long do children retain the acquired first aid knowledge?

By examining these research questions, we seek to shed light on the potential of schoolteachers as educators in fostering first aid knowledge among young children. The findings of this study may contribute to the development of comprehensive and age-appropriate first aid education programs, ultimately empowering children to become confident and competent first responders in times of emergency.

## 2. Materials and Methods

**Figure 1:**
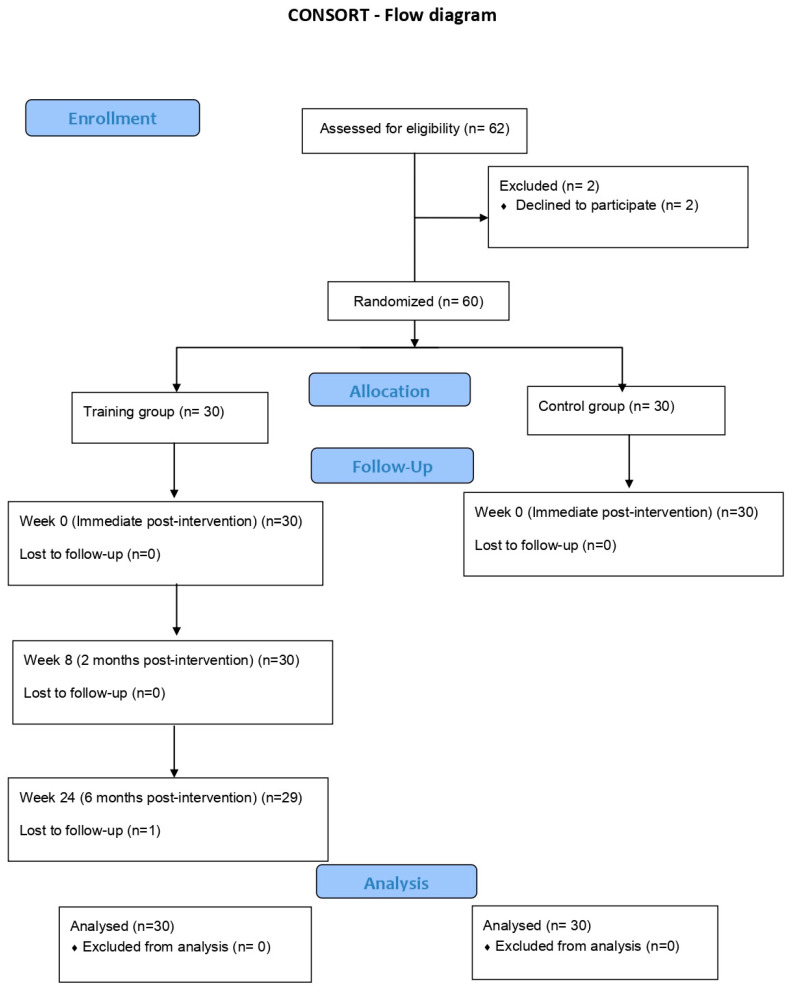
CONSORT diagram.

An experimental study with educational intervention was conducted in a randomly selected primary school, involving children in the 6–8 years age group. Prior to their participation, written informed consent was obtained from the parents or guardians of the children. The participants were divided into two groups: the training group and the control group (Figure 1). All children in the training group received a comprehensive first-aid course consisting of three lessons. These lessons were conducted by their regular schoolteachers, who were actively involved in the follow-up evaluations.

To assess the children’s knowledge and understanding of first aid, a structured questionnaire was administered to the training group one day prior to the commencement of the course, one day after completing the course, as well as at two months and six months post-training. The questionnaire, which encompassed twelve questions, including demographic information and ten questions focused on first aid scenarios, consisted of eight multiple-choice questions and two questions involving sequencing [9]. It is worth noting that adherence to the confidentiality regulations of the Greek national education system ensured the restricted access of researchers to the children’s personal data. Consequently, the study data are presented in the form of percentages with corresponding 95% confidence intervals, while descriptive statistics are presented as percentages and means.

Statistical analysis was performed using independent *t*-tests to compare the control group and the intervention group, and paired *t*-tests were employed to compare the pre-training, post-training, and follow-up results within the training group (significance level: *p* < 0.05). Additionally, it is important to mention that the study has been registered as a clinical trial under the identifier NCT05563129 since 3 October 2022.

### 2.1. Ethical Considerations

The University of Ioannina Ethics committee granted approval for this study (26 November 2021), ensuring that the research adheres to ethical guidelines and principles. Prior to the commencement of the study, comprehensive information was provided to the participating children, their parents, and the involved schoolteachers, both in written and oral formats. The researcher ensured that all parties involved were well-informed about the nature and purpose of the study. In particular, the parents of the children were informed about their rights, including the option to withdraw their child from the study at any time, without the need to provide a reason, and with complete anonymity. This approach aimed to prioritize the welfare and comfort of the children and their families, ensuring that their participation was voluntary and based on informed consent. To formalize their agreement for their child’s participation in the study, the parents were requested to provide written informed consent, indicating their understanding and willingness to involve their child in the research.

By following these ethical procedures and obtaining informed consent, the study ensures transparency, respect for autonomy, and protection of the rights and well-being of all participants involved.

### 2.2. Training Program

The training program implemented in this study encompassed a comprehensive approach, incorporating both theoretical components in the form of e-presentations and practical elements through role-playing exercises. Each lesson had a duration of 45 min, and the training spanned across three consecutive days. The responsibility of delivering the course fell upon two experienced classroom teachers. The training curriculum covered various essential topics related to first aid. The initial lessons aimed to familiarize the children with the meaning and significance of first aid, emphasizing its role in providing immediate assistance during emergencies. The main rules of first aid were also taught, equipping the children with a foundational understanding of how to respond appropriately in different situations.

One crucial aspect addressed in the training program was phoning an ambulance. The children were educated on the correct phone number to call for emergency medical services and were taught when it is necessary to contact an ambulance. Furthermore, they were guided on providing the vital information required to ensure prompt and efficient medical assistance. The training program also addressed specific injury scenarios commonly encountered by children. Topics such as head trauma, nose bleeding, choking, and bleeding were covered in detail, enabling the children to develop the necessary skills to respond effectively to such incidents.

To ensure the teachers’ proficiency in delivering the training, they received a comprehensive 2-h first aid training themselves. This additional training equipped them with the knowledge and expertise needed to effectively engage with the children and facilitate their learning.

Crucially, the training program was tailored to the age group of the participating children, considering the types and frequencies of accidents they are likely to encounter in their everyday lives [10]. This approach ensured that the training content was relevant, relatable, and practical, enhancing the children’s understanding and retention of the first aid concepts.

## 3. Results

The study encompassed a sample size of 60 children, accounting for approximately 35% of the total school population. These children belonged to the 6–8 years age group, with a distribution of 34 girls and 26 boys. On average, the participants had an age of 7.22 years. To assess the effectiveness of the training program, the knowledge of the children in the training group was evaluated at multiple time points. The evaluation process involved the administration of a study questionnaire, which served as a reliable tool for measuring the children’s understanding of first aid concepts. The first evaluation was conducted one day before the training course to establish a baseline of their existing knowledge. Subsequent evaluations were carried out one day after the completion of the course, as well as at two and six months after the training program concluded.

During the six-month evaluation, one child was absent, resulting in the completion of the questionnaire by 29 children. This robust evaluation process allowed for the analysis of knowledge retention and the long-term impact of the training program. In contrast, the control group, comprising children who did not receive the training, underwent a single evaluation at the beginning of the study. This evaluation served as a benchmark for comparing the knowledge levels between the trained group and the control group, highlighting the impact of the training program on the children’s understanding of first aid concepts. Table 1 presents a comprehensive comparison of the evaluation scores, enabling a visual analysis of the differences between the groups and the progression of knowledge before and after the training course.

The comparison between the trained and untrained children (control group) prior to the training revealed no significant difference in their initial levels of first aid knowledge. However, one day after the completion of the first aid course, the children in the training group demonstrated a significant improvement (*p* < 0.05) in their scores compared to the control group. Over the subsequent two and six months following the training, a gradual decline in knowledge was observed among the trained group. Despite this decline, their scores remained higher than both their pre-training scores and those of the control group, indicating a sustained advantage in first aid knowledge retention. Immediately after receiving the first aid training, the majority of children in the training group displayed a solid grasp of key concepts such as phoning an ambulance (including the correct phone number, when to call, and how to provide essential information), as well as dealing with head injuries, nose bleeding, bleeding, and choking. These areas of knowledge were particularly well-retained by the children in the training group. However, after the six-month period, there was a notable decrease in their knowledge regarding the handling of nose bleeding and trauma, as well as the proper actions to prevent choking. Table 2 provides an overview of the results, presenting the scores from the pre-training evaluation, the immediate post-training assessment, and the evaluations conducted at two and six months following the training.

## 4. Discussion

Our pilot study yielded a significant finding, demonstrating that children between the ages of 6 and 8 can acquire fundamental first aid principles. These principles encompassed crucial aspects such as understanding how to call an ambulance (including the correct phone number, the appropriate circumstances for contacting emergency services, and providing accurate information), as well as addressing head injuries, nosebleeds, bleeding from injuries, and choking. These findings align with previous research, which has also indicated that young schoolchildren are capable of providing first aid [8,9,10].

While the children’s performance on the first aid knowledge test declined at the two- and six-month follow-up assessments compared to their immediate post-training scores, their scores remained higher than their pre-training levels and those of the control group. This observation aligns with the findings reported by Banfai et al. [4,11]. Although there was some decay in knowledge retention over time, the overall improvement compared to the pre-training period underscores the lasting impact of the training program.

Notably, our study also emphasized the importance of addressing head injuries and trauma management in the primary school curriculum. Head injuries are a common occurrence reported within school settings [12], yet there is a dearth of research examining the inclusion of head injury and trauma management in primary school curricula. Previous studies have predominantly focused on basic life support (BLS) instruction. However, studies have consistently shown that children under the age of ten struggle to perform proper chest compressions for BLS [13,14]. Consequently, our first aid training course for 6–8-year-olds did not incorporate BLS instruction.

In an effort to optimize the effectiveness and cost efficiency of our intervention, we enlisted the children’s regular schoolteachers to conduct the first aid program. While many studies have employed specialized first aid instructors [15,16], a few studies have demonstrated positive outcomes when children are trained in first aid by their own schoolteachers [17,18]. If schoolteachers can achieve comparable results to specialized instructors, the administration of first aid lessons by schoolteachers becomes a more financially viable approach. Moreover, our teaching strategy adopted a blended approach, as research has established that scenario-based methods incorporating both practical and theoretical components enhance the learning experience for primary school children [19].

Teaching first aid to children is a crucial step in fostering their responsibility and safety as active members of society [20]. Equipping children with basic first aid skills empower them to react promptly and calmly during emergencies, potentially saving lives and preventing serious injuries. First aid education can be tailored to different age groups, and even young children can grasp essential skills such as calling for help and positioning someone in the recovery position [18,20,21]. As children grow older, they can acquire more advanced skills such as CPR and the use of automated external defibrillators (AEDs) [22,23]. By instilling these skills from an early age, we cultivate a culture of preparedness and empower our children to become capable responders in times of crisis.

Children may have a basic awareness of certain parts of first aid prior to getting professional first aid instruction. They may be aware of how to summon assistance in an emergency or where to find an adult if someone is harmed. They may be knowledgeable of bandaging and using ice packs to treat small wounds. Their knowledge, however, may be insufficient and lack the depth and accuracy needed to provide good support. They may be unaware of CPR, choking control, and how to respond to diverse injuries [3,8,9]. For this reason, we anticipated that the questions “Correct reaction to nose bleeding”, “Correct actions to prevent choking”, “Correct actions to call the ambulance”, and “Correct reaction to trauma” would have had less correct answers.

There are several reasons why teaching first aid to children is important [6]. First and foremost, it can help children develop a sense of responsibility and empowerment. By learning how to respond to emergency situations and provide first aid, children can feel more confident and capable of handling unexpected situations. This can boost their self-esteem and help them develop a sense of self-efficacy. In addition, teaching first aid to children can help promote safety awareness and prevent accidents and injuries from occurring in the first place. Children who are aware of potential hazards and know how to respond to emergencies are less likely to be injured or become involved in dangerous situations. Moreover, teaching first aid to children can help promote empathy and compassion [6,9]. By learning how to care for injured or ill individuals, children can develop a deeper understanding of the needs and feelings of others. This can help promote positive social and emotional development and foster a sense of community and caring.

When teaching first aid to children, it is important to use age-appropriate methods and materials [18,23]. Younger children may benefit from interactive games and activities that teach basic safety and first aid skills, while older children may be able to learn more advanced techniques and procedures. One effective way to teach first aid to children is through experiential learning [24,25]. This involves providing hands-on opportunities for children to practice first aid skills and respond to simulated emergency situations. This can help reinforce learning and increase children’s confidence and readiness to respond to real-life emergencies. In conclusion, teaching first aid to children can be integrated into existing curricula and programs, such as physical education, health education, and after-school clubs. This can help ensure that first aid education is accessible and available to all children, regardless of their background or circumstances.

We believe that the first aid program should be made mandatory in schools. Young children can learn first aid and their schoolteachers can teach first aid [25,26]. Because the young children in our study had forgotten some first aid knowledge six months after the training, we recommend that the program should be repeated once a year.

## 5. Limitations

While this study provides valuable insights into the effectiveness of the first aid training program for young children, it is important to acknowledge certain limitations that should be taken into consideration when interpreting the results. One limitation of the study is the relatively small sample size. Although the study included 60 children, representing approximately 35% of the total school population, a larger sample size would have provided a more robust and representative dataset. A larger sample would have allowed for a more comprehensive analysis and increased generalizability of the findings. Another limitation is the lack of ongoing assessment for the control group over the six-month period. Since all participants were from the same school, it is plausible that the control group may have acquired some knowledge incrementally during this time. Without reassessment, it is challenging to accurately compare the progress and knowledge retention between the trained and untrained children throughout the study period. Furthermore, the study did not undergo formal validation, which may limit its applicability to other populations. Validation studies are crucial in establishing the reliability and generalizability of research findings across different settings, age groups, and cultural contexts. Therefore, caution should be exercised when extrapolating the results of this study to other populations or educational settings. These limitations highlight the need for future research to address these shortcomings. Studies with larger sample sizes, longitudinal assessments of control groups, and validation in diverse populations would contribute to a more comprehensive understanding of the effectiveness and generalizability of first aid training programs for young children.

## 6. Conclusions

First aid is an essential skill that empowers individuals to provide immediate assistance during emergencies. By instilling a culture of first aid from childhood, we can cultivate a society where people possess the aptitude and confidence to respond effectively in crisis situations [24,25]. Starting early, children should be exposed to basic first aid knowledge in a simple and age-appropriate manner [20,24,25]. Schools can incorporate first aid training into their curriculum, teaching students how to recognize common injuries, perform CPR, and administer basic care. Additionally, interactive workshops and simulations can be organized, encouraging active participation and hands-on learning. We can develop a culture of first aid from childhood by providing first aid education in schools, involving families in training, and supporting community activities. Empowering people with this knowledge and ability will result in a safer society where people are confident and capable of assisting when it matters the most.

This pilot study aimed to assess the effectiveness of teaching first aid to 6–8-year-old children in primary school, delivered by their own schoolteachers. The findings of the study present promising outcomes, indicating that children within this age group have the capacity to grasp basic first aid principles, including knowing how to call an ambulance (including the correct phone number, when to seek emergency assistance, and how to provide accurate information), as well as effectively addressing common scenarios such as head injuries, nosebleeds, bleeding from injuries, and choking, when instructed by their schoolteachers. While the children’s performance on the first aid knowledge test declined at the two- and six-month follow-up assessments compared to their immediate post-training scores, their scores remained superior to their pre-training levels and those of the control group. This observation suggests that the scenario-based training implemented in the study effectively developed the children’s first aid knowledge, and that they retained a significant portion of this knowledge even six months after the training.

This study sheds light on the potential of incorporating scenario-based first aid and trauma management training in primary schools. By developing children’s first aid knowledge from an early age, we equip them with valuable life skills that can make a significant difference in emergency situations. Further research and advancements in this field will undoubtedly contribute to refining the curriculum and methodology for teaching young children about trauma management, ensuring their preparedness to respond effectively in real-life scenarios.

## Figures and Tables

**Table 1:** Difference in first aid knowledge between the control group (*n* = 30) and the first aid training group (*n* = 30) of 6–8-year-old primary school children.

Group Comparison	*p* Value	Median
Control vs. Training (pre-test) (independent *t*-test)	0.4422	60 vs. 56.5
Control vs. Training (post-test) (independent *t*-test)	0.0002	60 vs. 97
Control vs. Training (post 2 months) (independent *t*-test)	0.0005	60 vs. 93.5
Control vs. Training (post 6 months) (independent *t*-test)	0.0065	60 vs. 89.5
Before-training vs. after-training at day 1 (paired *t*-test)	0.0003	56.5 vs. 97
Before-training vs. follow-up at 2 months (paired *t*-test)	0.0003	56.5 vs. 93.5
Before-training vs. follow-up at 6 months (paired *t*-test)	0.0001	56.5 vs. 89.5
After-training at day 1 vs. follow-up at 2 months (paired *t*-test)	0.0953	97 vs. 93.5
After-training at day 1 vs. follow-up at 6 months (paired *t*-test)	0.0146	97 vs. 89.5

**Table 2:** Correct answers (%) on first aid questionnaire of 6–8-year-old children in the first aid training group (*n* = 30) and the control group (*n* = 30).

Topic	Activity	Pre-Test	Post-Test	After 2 Months	After 6 Months	Control Group
Phoning the ambulance	Correct emergency number	83	97	97	93	90
	Correct cause for calling the ambulance	90	97	100	97	87
	Correct information to the rescuers	60	93	87	100	67
	Correct order in calling the ambulance	33	93	83	79	37
Manage bleeding	Correct management of nose bleeding	23	100	83	65	27
	Correct management of minor bleeding	73	100	100	100	70
Choking	Correct management of choking	53	100	97	93	53
	Correct order to prevent choking	10	90	77	55	17
Injury	Correct management of head trauma	80	97	97	86	77
	Correct management of trauma	7	80	90	62	7

## Data Availability

No other data are available.
